# Explaining Productivity Differences Among Tree Species via Biotic and Abiotic Factors

**DOI:** 10.3390/life16020277

**Published:** 2026-02-05

**Authors:** Liyang Tong, Kai Chen, Xiahuan Zhan, Kai Wang, Huajing Song, Li Ma, Lijin Wang

**Affiliations:** 1 College of Materials and Energy Engineering, Lishui University, Lishui 323000, China; 2 College of Agriculture and Biotechnology, Lishui University, Lishui 323000, China; 3 Zhejiang Lishui Songyang County Environmental Monitoring Station, Lishui 323400, China

**Keywords:** climate change, *Cunninghamia lanceolata*, *Pinus massoniana*, biomass, biodiversity, forest structure, forest management

## Abstract

Greenhouse gases emitted by humans have exacerbated global climate change. Forests can effectively sequester atmospheric carbon dioxide through photosynthesis, and afforestation has been widely adopted worldwide to mitigate climate change. *Cunninghamia lanceolata* and *Pinus massoniana*, as major afforestation tree species, are extensively cultivated in southern China. However, the mechanisms by which climate, topography, biodiversity, forest structure, and forest growth status affect the productivity of these two species remain unclear. This study used forest inventory data from Lishui City combining the Biomod2 model with a structural equation model (SEM) to investigate the differential effects of biotic and abiotic factors on the productivity of the two tree species. The results showed that at the same diameter at breast height (DBH), the biomass of *P. massoniana* reached 384.67 kg, accounting for 188.75% of that of *C. lanceolata* (211.07 kg). The dominant climatic factors affecting *C. lanceolata* and *P. massoniana* were different; the most important climatic factors affecting *C. lanceolata* were Bio 17, Bio 15, Bio 05, Bio 08, and Bio 02, while those affecting *P. massoniana* were Bio 18, Bio 04, and Bio 01. Furthermore, the explanatory power of the structural equation model (SEM) optimized by the Biomod2 model was effectively improved. Biodiversity and forest growth factors were the most important biotic factors affecting *C. lanceolata* (*p* < 0.01), while structural diversity and forest growth factors were the most important biotic factors affecting *P. massoniana* (*p* < 0.05). Biodiversity and structural diversity exerted divergent effects on *C. lanceolata* and *P. massoniana* in different growth stages, exerting negative effects in the early growth stage and positive effects in the late growth stage. These outcomes were jointly driven by the selection effect and niche complementarity. This study recommends the forest management practices should select tree species based on local conditions.

## 1. Introduction

Combustion of fossil fuels has led to an increase in the atmospheric concentrations of various greenhouse gases including CO_2_, exacerbating global climate change and posing new challenges to forest ecosystems [1,2,3]. Forests can effectively sequester atmospheric CO_2_ through photosynthesis. As the main component of terrestrial carbon sequestration, they are crucial for regulating atmospheric carbon concentrations [4]. Expanding forest areas will effectively reduce atmospheric greenhouse gas levels and mitigate the impacts of climate change [5]. Currently, countries worldwide are adopting afforestation measures to address climate change. The global area of planted forests increased by more than 1.05 × 10^8^ ha from 1990 to 2015, reaching a total area of 2.91 × 10^8^ ha [6]. It is projected that China’s forest coverage rate will rise to 26% by 2050 [7]. From the perspective of forest management, the approaches to effectively enhance forest productivity remain unclear. Biotic and abiotic factors may give rise to low-efficiency forests, resulting in the waste of resources [8,9,10]. Therefore, assessing the relationship between environmental conditions and biomass is of great significance for forest management and the efficient utilization of resources.

The relationship between forest diversity and forest productivity is mainly driven by abiotic and biotic factors, among which the abiotic include climate and topography [11,12,13]. Forest productivity at different scales exhibits divergent responses to environmental conditions, climate acts as the dominant environmental factor in large-scale forests [14], and topography exerts more pronounced effects in small-scale forests [12,15]. Biotic factors encompass biodiversity, structural diversity, and forest growth factors [3,16,17]. The linkages among these factors are primarily mediated by two mechanisms, namely niche complementarity and the selection effect [18,19]. Niche complementarity refers to the phenomenon where differences in species niches enable more efficient resource utilization, thereby further enhancing resource use efficiency and forest productivity [20]. The selection effect describes the scenario in which dominance by species with high productivity or key ecological functions exerts positive impacts on the productivity of forest ecosystems [21]. The combined effects of these factors regulate the productivity of trees in forest ecosystems. However, the underlying mechanisms governing this phenomenon remain unclear, thus warranting further investigation.

*Cunninghamia lanceolata* and *Pinus massoniana* are the most common coniferous tree species in southern China. They have been widely adopted as ideal afforestation species, owing to their rapid growth rate, strong adaptability, and considerable economic and ecological benefits [22]. At present, the plantation areas of *C. lanceolata* and *P. massoniana* in southern China have reached as large as 9.90 × 10^6^ ha and 2.51 × 10^6^ ha, respectively. Large-scale afforestation projects, however, may give rise to a series of ecological problems, including simplified forest composition, water resource scarcity, and intensified conflicts between farmland and forest land in some regions [7,23]. Notably, the inherent biological traits of plants result in distinct ecological requirements across different growth stages, such as those associated with root systems [15,24] and altitudinal gradients [25,26]. These ecological requirements, in conjunction with environmental conditions and forest diversity, jointly regulate tree productivity [26]. Understanding the mechanisms underlying these combined effects is crucial for enhancing forest productivity. Therefore, it is imperative to further elucidate these interactive mechanisms.

This study focuses on the impacts of climate, topography, biodiversity, structural diversity, and forest growth factors on the productivity of *C. lanceolata* and *P. massoniana*, while exploring in depth their actual effects and the underlying response mechanisms. Two hypotheses are proposed in this study: (1) Topography and climate exert differential impacts on the growth of *C. lanceolata* and *P. massoniana*. (2) The ecological mechanisms governing the biomass of *C. lanceolata* and *P. massoniana* are distinct. By centering on the effects of biotic and abiotic factors on conifer productivity, this study aims to provide scientific guidance for the efficient and sustainable utilization of forest resources.

## 2. Materials and Methods

### 2.1. Study Area and Field Work

**Figure 1:**
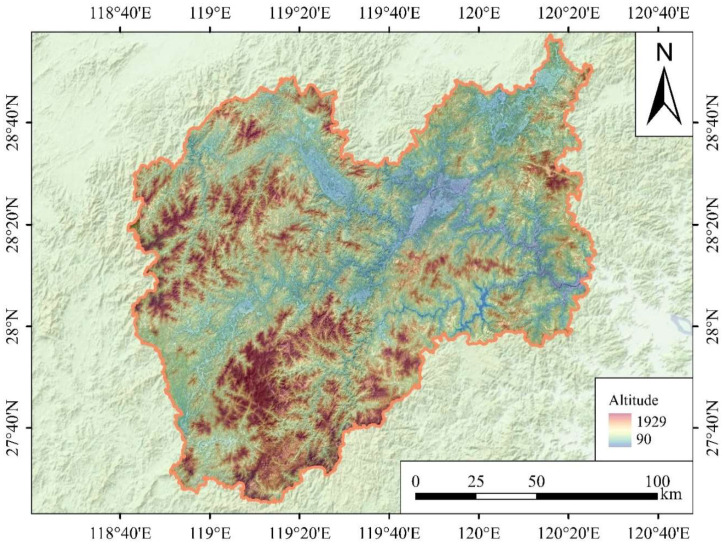
Study area.

Lishui City, Zhejiang Province, is located in the southwestern part of Zhejiang Province, with geographic coordinates ranging from 27°25′ to 28°57′ N latitude and from 118°41′ to 120°26′ E longitude. The landform of this region is dominated by mountainous areas featuring high mountains and steep slopes, and the terrain slopes downward from the southwest to the northeast. The highest peak in Zhejiang Province is situated in Lishui City, with a maximum altitude of 1929 m (Figure 1). The total area of Lishui City is 1.73 × 10^4^ km. It falls within the mid-subtropical monsoon climate zone, characterized by a warm and humid climate, abundant precipitation, and typical mountain climate features. Such topographic and climatic conditions endow Lishui with abundant biodiversity resources.

The data used in this study were derived from the forest resource survey conducted in Lishui City from August to October 2024, in which the surveyed forest types included coniferous forests, broad-leaved forests, and mixed coniferous–broad-leaved forests. To investigate the current status of biomass accumulation of *C. lanceolata* and *P. massoniana* under different natural environments, this study selected forest plots containing these two species, including 85 plots of *C. lanceolata* and 54 plots of *P. massoniana*. All of these plots were distributed across different natural habitats (Table 1). Each forest plot had a size of 20 m × 20 m. In each plot, we recorded the following indicators of woody plants with a diameter at breast height (DBH) ≥ 5 cm: species, DBH (cm), tree height (TH, m), and crown length (CW, m).

### 2.2. Biomass and Diversity Indices

The allometric equation method was adopted in this study to separately calculate the stem biomass, root biomass, crown biomass, and total biomass of *C. lanceolata* and *P. massoniana* (kg, Table S1) [27]. In order to facilitate the statistical analysis of data, the DBH of standing trees was classified into multiple diameter classes at intervals of 5 cm for segmented analysis [28,29]. The natural-break classification method (Jenks) was used to identify the DBH corresponding to the critical points of biomass variation in *C. lanceolata* and *P. massoniana* [30].

The Shannon–Wiener index (H) was employed to quantify the species diversity across different plots [3]. The coefficient of variation in tree height (CV_TH_, %) within each plot was selected to represent the structural diversity [3,31]. The basal area (BA) of trees in each plot was used to indicate the forest growth index [16,32].

(1) $$H=-\sum \left(Pi\right)\left(\ln Pi\right)$$

(2) $$BA=\pi {\left(\frac{DBH}{2}\right)}^{2}/400$$

where *Pi* indicates the proportion of individuals of a given species relative to the total number of individuals in the community.

### 2.3. Climate Variable Screening and Topographic Factors

Climate is an important factor affecting plant growth. In this study, climatic variables were derived from WorldClim (http://www.worldclim.org), which includes 19 climatic variables (Table S2). This study employed the Biomod2 species distribution model to screen climatic variables and construct the potential distribution areas of *C. lanceolata* and *P. massoniana* in Lishui City. The detailed methods for model construction are provided in the Supplementary Material. To avoid high multicollinearity in the model, pre-modeling processing was first performed for *C. lanceolata* and *P. massoniana* to obtain the initial contribution of each factor. Pearson correlation analysis of climatic factors was conducted using R software (Figure S1). Based on the results of the initial model contribution and Pearson correlation analysis, factors with a correlation coefficient |r| ≥ 0.7 were excluded to reduce model multicollinearity [33]. The screened climatic factors were used to construct the final Biomod2 model, and the most important climatic factors output by the final ensemble model were selected for subsequent analyses.

Topographic data were recorded using RTK (Real-Time Kinematic, South Surveying and Mapping, Guangzhou, China) instruments in the sample plots, including altitude, slope gradient, and slope aspect (Table 1).

### 2.4. Statistical Analysis and Structural Equation Model

Linear regression and least-significant-difference (LSD) tests were used to assess the statistical significance of environmental effects on biomass (*p* < 0.05). Principal component analysis (PCA) was employed for dimensionality reduction in environmental factors, and the dimensionality-reduced data were used for subsequent analyses. In this study, the “bestnormsize” package was utilized to improve the normality of the data, and *Z*-score transformation was performed on all variables to facilitate the interpretation of parameter estimates on a comparable scale.

Based on the variable relationships among environmental factors (climate and topography), species diversity (H), stand structural diversity (CV_TH_), the forest growth index (BA), and species biomass [34], the “piecewiseSEM” package was used to construct separate structural equation models (SEMs) for the two species. The fit criteria for the SEMs were defined as follows: *p* > 0.05, a goodness-of-fit index (GFI) > 0.9, a comparative fit index (CFI) > 0.95, and a standardized root mean square residual (SRMR) < 0.08. When these criteria were satisfied, the model was considered to have good statistical validity [3]. All plotting and statistical analyses were conducted using R (v 4.3.1).

## 3. Results

### 3.1. Forest Biomass

**Figure 2:**
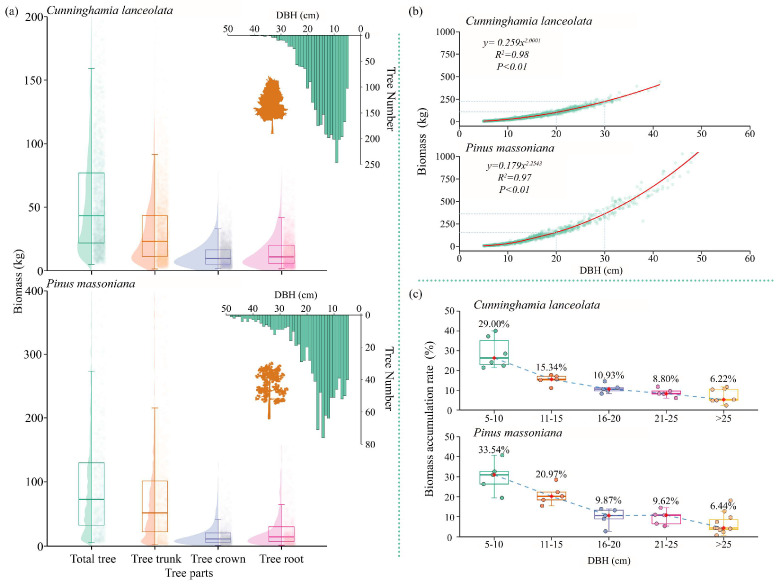
Relationship diagram of DBH and biomass in *C. lanceolata* and *P. massoniana*. (**a**) Biomass allocation by organs and DBH histogram for *C. lanceolata* and *P. massoniana*; (**b**) relationship between DBH and biomass of *C. lanceolata* and *P. massoniana*; (**c**) statistical chart of biomass accumulation rate for *C. lanceolata* and *P. massoniana*. Biomass accumulation rate: rate of increase in biomass per 5 cm increase in DBH.

The results of this study showed that there were significant differences in biomass and the accumulation rate between the two coniferous species. The total biomass of *P. massoniana* was higher than that of *C. lanceolata* (Table 2, Figure 2b). The biomass of *P. massoniana* with a DBH of 25–30 cm was 384.67 kg, which was 188.75% of the biomass of *C. lanceolata* with the same DBH (211.07 kg). The DBH distributions of both *P. massoniana* and *C. lanceolata* followed a J distribution (Figure 2a). *P. massoniana* had a higher total biomass accumulation, with an average of 231.79% that of *C. lanceolata*. Additionally, *P. massoniana* exhibited a higher biomass accumulation rate (Figure 2c). Specifically, when DBH ranged from 11 to 15 cm, the biomass accumulation rate of *P. massoniana* was 5.36% higher than that of *C. lanceolata*, leading to its higher overall biomass—this result is consistent with that in Figure 2a. For both tree species, the coefficient of variation in biomass was larger at a small DBH and gradually decreased with tree growth, indicating a trend toward more stable growth. Jenks results showed that the critical DBH values corresponding to biomass variation in *C. lanceolata* and *P. massoniana* were 11 cm and 10 cm, respectively (Figure S2). This study hypothesizes that there may be a certain threshold effect during the growth of the two tree species, and their response factors to the environment are variable; subsequent studies will be conducted based on this hypothesis.

### 3.2. Environmental Factors and Forest Diversity

**Figure 3:**
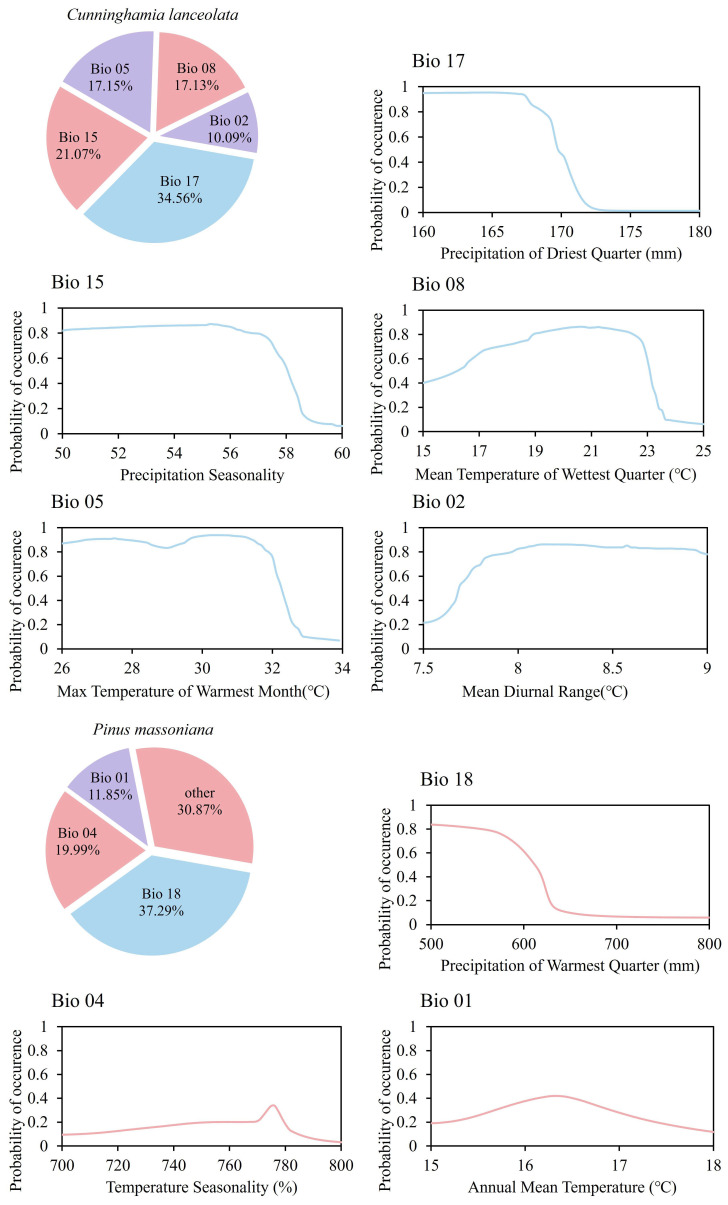
Factor contribution degrees and response curve diagrams of *C. lanceolata* and *P. massoniana*.

The Biomod2 ensemble model exhibited good fitting performance, with the area under the curve (AUC) and the true skill statistics (TSS) higher than those of single models; therefore, the ensemble model was reliable (Table S3). Model results indicated that the primary factors influencing the distribution of *C. lanceolata* were Bio 17 (34.56%), Bio 15 (21.07%), Bio 05 (17.15%), Bio 08 (17.13%), and Bio 02 (10.09%). For *P. massoniana*, the dominant distribution-influencing factors were Bio 18 (37.29%), Bio 04 (19.99%), and Bio 01 (11.85%), while the contribution rates of the remaining factors were all below 10%. Therefore, this study selected these climatic factors as predictor variables for subsequent analyses (Figure 3).

The Biomod2 ensemble model results showed that the suitable areas of *C. lanceolata* and *P. massoniana* were 9809.90 km^2^ and 7514.12 km^2^, respectively (Figure S4). *C. lanceolata* had a larger suitable area, with the difference mainly distributed in the southwestern part of Lishui City.

**Figure 4:**
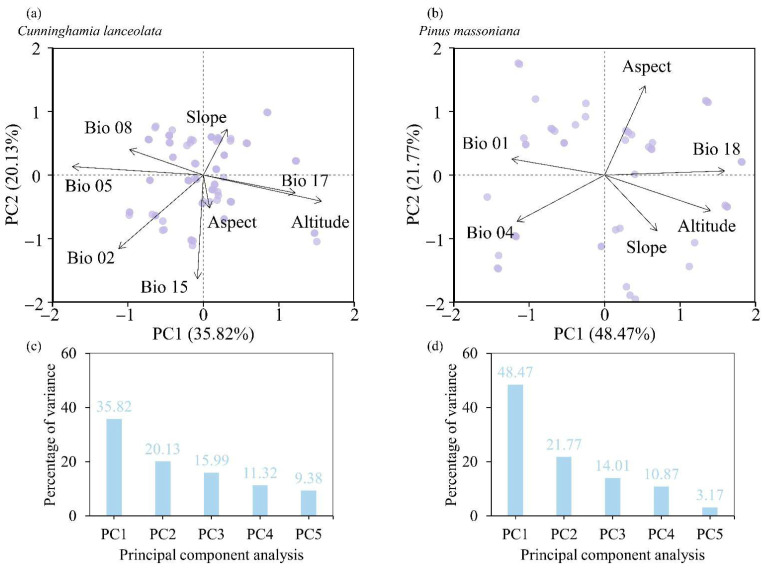
Principal component analysis (PCA) plot of environmental factors. (**a**) Environmental factors in *C. lanceolata*; (**b**) environmental factors in *P. massoniana*; (**c**) PCA percentage of variance in *C. lanceolata*; (**d**) PCA percentage of variance in *P. massoniana*.

Principal component analysis (PCA) was performed to analyze environmental factors (Figure 4a). The results indicated that the cumulative explanatory power of the two axes for *C. lanceolata* and *P. massoniana* was 55.95% and 70.25%, respectively (Figure 4b). The first axis (environmental PC1, Env PC1) and the second axis (environmental PC2, Env PC2) of the PCA were used as predictor variables for subsequent analyses (Table 3).

**Figure 5:**
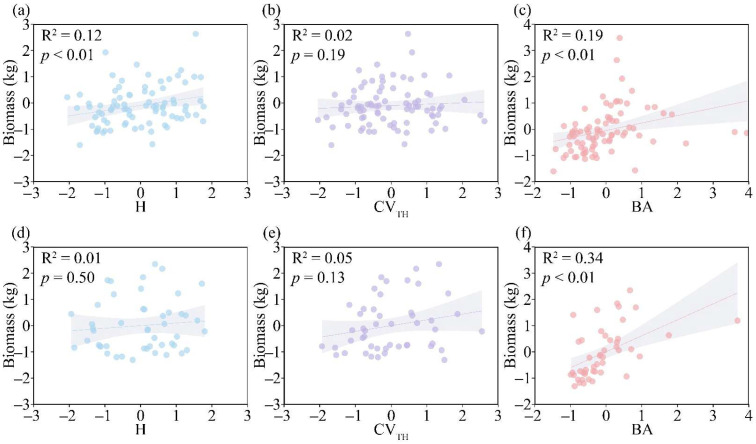
The relationship between the species diversity, structural diversity, basal area, and biomass of two coniferous species. (**a**) The relationship between the species diversity and biomass of *C. lanceolata*. (**b**) The relationship between the structural diversity and biomass of *C. lanceolata*. (**c**) The relationship between the basal area and biomass of *C. lanceolata*. (**d**) The relationship between the species diversity and biomass of *P. massoniana*. (**e**) The relationship between the structural diversity and biomass of *P. massoniana*. (**f**) The relationship between the basal area and biomass of *P. massoniana*. H, Shannon–Wiener index; BA, forest basal area. CV_TH_, coefficient of variation in tree height.

Forest diversity exerted different effects on the biomass of the two coniferous species. Specifically, biodiversity (H) showed a significant positive correlation with the biomass of *C. lanceolata* (Figure 5a; *p* < 0.01) but had no significant effect on that of *P. massoniana* (Figure 5d). Structural diversity (CV_TH_) exerted positive effects on the biomass of both *C. lanceolata* and *P. massoniana* (Figure 5b,e). Forest growth factors exhibited significant positive effects on the biomass of both tree species (Figure 5c,f; *p* < 0.01). These results indicated that the three biotic factors collectively promoted the biomass of both species. In this study, we incorporated forest factors and environmental factors to further analyze the driving factors.

### 3.3. Driving Factor Analysis of Forest Biomass

**Figure 6:**
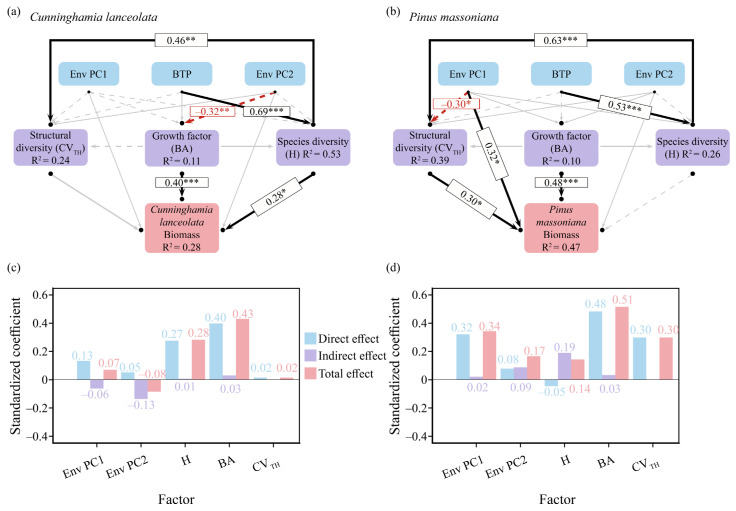
Structural equation model after optimization presenting the effects of environmental variables, broad-leaved tree proportion, plant species diversity, forest basal area, and stand structural diversity on biomass. (**a**) Path diagrams of factors influencing changes in *C. lanceolata* biomass; (**b**) path diagrams of factors influencing changes in *P. massoniana*. (**c**) Total direct and indirect effects combined in *C. lanceolata* biomass. (**d**) Total direct and indirect effects combined in *P. massoniana* biomass. Env PC1, environmental PC1; Env PC2, environmental PC2; H, Shannon–Wiener index; BTP, broad-leaved tree proportion; BA, forest basal area. CV_TH_, coefficient of variation in tree height. All variables were Z-score-transformed prior to analysis. *: *p* < 0.05,**: *p* < 0.01,***: *p* < 0.001.

The structural equation models (SEMs) for the biomass of *C. lanceolata* and *P. massoniana* both exhibited good fitting performance (Table S5). The SEM constructed using mean annual temperature and mean annual precipitation as environmental factors explained 27% and 44% of the biomass variation in *C. lanceolata* and *P. massoniana*, respectively (Figure S4). In contrast, the SEM constructed using the screened environmental factors accounted for 28% and 47% of the biomass variation in the two species, respectively (Figure 6a,b). The explanatory power of the optimized model was improved; thus, the optimized SEM was adopted for all subsequent analyses.

The most significant factors affecting the biomass of *C. lanceolata* were H and BA, whereas the most significant factors influencing the biomass of *P. massoniana* were Env PC1, CV_TH_, and BA. All these factors exerted promoting effects on biomass, and the results were consistent with those of the linear regression analysis. The variable effect analysis plots revealed the direct effects, indirect effects, and total effects between the factors and biomass. Environmental factors exerted positive direct and total effects on the biomass of *C. lanceolata* but negative indirect effects. In contrast, structural diversity, species diversity, and forest growth factors all showed positive effects on the biomass of *C. lanceolata* (Figure 6c). All factors exhibited positive effects on the biomass of *P. massoniana*.; however, H had a weak negative direct effect, with its total effect being positive (Figure 6d). These results indicated that forest growth factors had substantial effects on the biomass of both species. Species diversity exerted a greater impact on the biomass of *C. lanceolata*, while structural diversity had a more pronounced effect on the biomass of *P. massoniana*.

**Figure 7:**
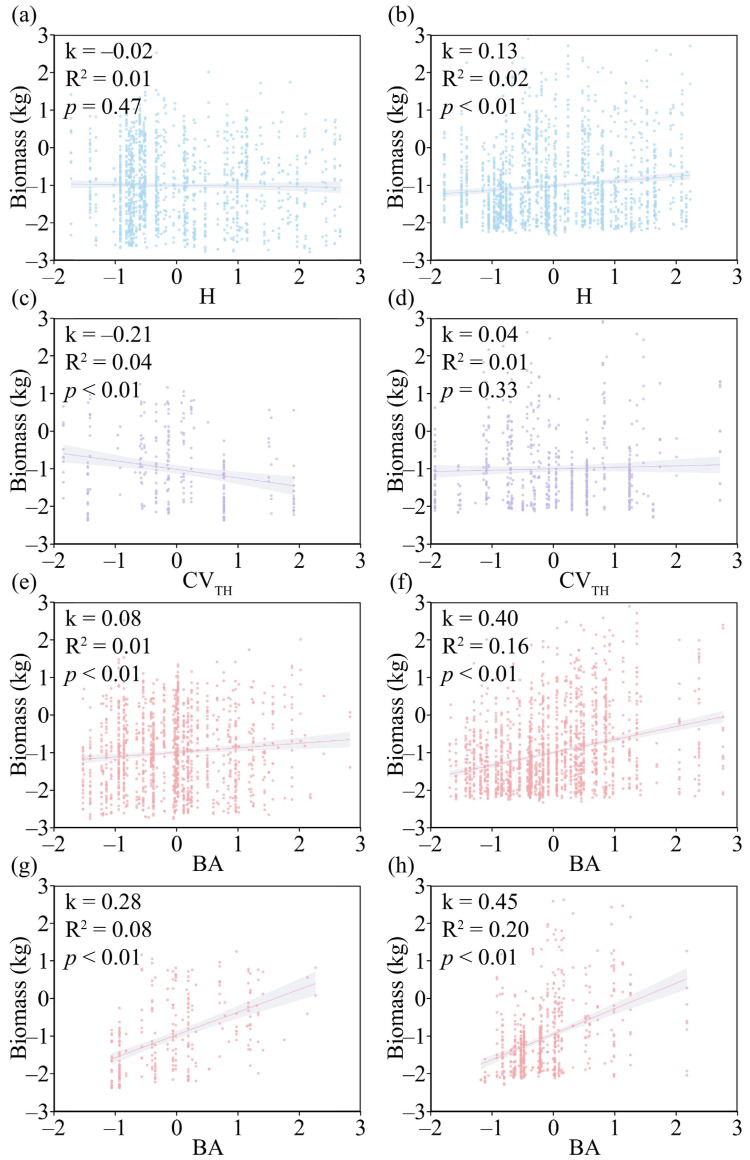
Responses of different growth stages of *C. lanceolata* and *P. massoniana* to biodiversity, structural diversity, and biological growth factors. (**a**) The impact of H on the biomass of *C. lanceolata* with a diameter at DBH less than 11 cm; (**b**) The impact of H on the biomass of *C. lanceolata* with a diameter at DBH greater than 11 cm; (**c**) The impact of CV_TH_ on the biomass of *P. massoniana* with a diameter at DBH less than 10 cm; (**d**) The impact of CV_TH_ on the biomass of *P. massoniana* with a diameter at DBH greater than 10 cm; (**e**) The impact of BA on the biomass of *C. lanceolata* with a diameter at DBH less than 11 cm; (**f**) The impact of BA on the biomass of *C. lanceolata* with a diameter at DBH greater than 11 cm; (**g**) The impact of BA on the biomass of *P. massoniana* with a diameter at DBH less than 10 cm; (**h**) The impact of BA on the biomass of *P. massoniana* with a diameter at DBH greater than 10 cm.

Forest diversity and forest growth factors exerted consistent effects on *C. lanceolata* and *P. massoniana* across different growth stages. In the early growth stage of *C. lanceolata*, H exerted a negative effect on its biomass (Figure 7a). In the middle and late growth stages, H showed a significant positive effect on its biomass (Figure 7b; *p* < 0.05). For *P. massoniana*, CV_TH_ exerted a significant negative effect on its biomass in the early growth stage (Figure 7c; *p* < 0.05), whereas CV_TH_ exerted a positive effect on its biomass in the middle and late growth stages (Figure 7d). BA exerted a significant promoting effect on both *C. lanceolata* and *P. massoniana* across all growth stages (Figure 7e–h; *p* < 0.05).

## 4. Discussion

### 4.1. Difference Analysis of Growth of C. lanceolata and P. massoniana

*C. lanceolata* and *P. massoniana* are the most common coniferous tree species in southern China, and they are recognized as ideal afforestation species due to their considerable economic and ecological benefits [22]. In this study, field survey data were used to quantify the biomass of *C. lanceolata* and *P. massoniana* in natural environments, with DBH serving as the independent variable to explore the relationship between DBH and biomass. Existing studies have demonstrated that tree age is significantly correlated with DBH [35]. Tree age is positively associated with DBH and biomass accumulation over time [36], which indicates that the direct use of DBH as an analysis indicator has good theoretical significance. In natural forests, trees of the same species with identical DBH may exhibit variations in age [37]. This is because trees adopt different resource utilization strategies during different forest developmental stages, leading to temporal variations in growth even within the same species [38]. In addition, obtaining tree increment cores is a destructive sampling approach. When sampling thousands of trees, even the advanced increment bore coring (IBC) technique may cause tree mortality [39], resulting in potential ecological damage. Therefore, this study focused on the relationship between DBH and biomass in natural environments based on DBH data, thereby avoiding the interference caused by tree age heterogeneity and destructive sampling, as well as the adverse impacts on forest ecosystems.

The results presented in Figure 2 show that the biomass and accumulation rate of *P. massoniana* were higher than those of *C. lanceolata*. This finding is inconsistent with the results of analyses conducted in southeastern (SE) China [40] but closely consistent with the data obtained from forests under near-natural conditions [41]. This discrepancy is attributable to differences in spatial scales and geographical regions, and the study area is characterized by extensive Karst landforms, dominated by mountain forests with steep slopes (Figure 1). *C. lanceolata* is a shallow-rooted plant. In steep-slope habitats, although the root biomass of *C. lanceolata* increases significantly, the low root–shoot ratio (R:S) of plants in subtropical environments causes most biomass to be allocated to above-ground parts [42]. The steep slopes contribute to reduced growth performance of *C. lanceolata* [15], thereby diminishing its biomass accumulation rate. In contrast, *P. massoniana* has a distinct taproot system, which enables it to grow well in rocky crevices and slopes with exposed bedrock [24]. The soil in the study area is acidic. *C. lanceolata* prefers loose, deep, humus-rich soils, whereas *P. massoniana* favors acidic soil conditions [40]. Owing to the interspecific differences in topographic and edaphic adaptations, *P. massoniana* exhibits a higher biomass accumulation rate.

### 4.2. The Model Optimization, and Effects of Environmental Factors on the Biomass of C. lanceolata and P. massoniana

Current studies typically adopt Bio 01 and Bio 12 as variables to investigate the effects of climatic conditions on forest productivity [3]. However, the study area of the present research is relatively small, so the actual impacts of these two climatic factors across the entire region may be limited [14]. In addition, different tree species exhibit divergent responses to climatic conditions, meaning that the same set of climatic factors cannot be used to explain the growth dynamics of different species [43,44]. As an ensemble model, Biomod2 has superior predictive ability compared to single models [33,45]. In this study, Biomod2 was employed to screen the most influential factors for *C. lanceolata* and *P. massoniana*, which were then used as the climatic variables for subsequent analyses. The SEMs presented in Figure 6 and Figure S4 were constructed using the screened factors and conventional climatic factors, respectively. The results showed that constructing SEMs with the screened climatic factors could improve the explanatory power (with an increase of 0.01 for *C. lanceolata* and 0.03 for *P. massoniana*), thus verifying the effectiveness of this method. Nevertheless, the explanatory power of the SEMs remained relatively low, probably due to the lack of soil property data, belowground biomass data, and long-term forest monitoring data. Previous studies have shown that the inclusion of soil property data can increase the model’s explanatory power to 51% [3]. Therefore, this study still has room for further improvement.

The results of the Biomod2 model showed that the climatic potential suitable area for *C. lanceolata* was larger and more extensive in the study area, and this is attributed to the effect of altitude. The results of the field survey indicated that the maximum recorded altitude of *C. lanceolata* reached 1141.35 m, whereas that of *P. massoniana* was only 667.29 m. Current studies have demonstrated that *C. lanceolata* has a higher altitudinal adaptability than *P. massoniana*, with a maximum altitude tolerance of up to 2200.00 m [26]. Although *P. massoniana* can grow well on steep slopes [24], the low mean temperature and winter frost at high altitudes result in a distinct altitudinal upper limit for this species (Figure 3) [25]. These results are consistent with the first hypothesis proposed in this study. Therefore, this study suggests that forest management practices should take into account the adaptability of tree species, which can effectively enhance the growth performance of the target species.

### 4.3. The Effects of Forest Factors on the Biomass of C. lanceolata and P. massoniana

The SEM results indicated that forest diversity and forest growth indices exert significant effects on the biomass of *C. lanceolata* and *P. massoniana*. Specifically, *C. lanceolata* exhibits significant responses to H and BA, whereas *P. massoniana* shows significant responses to CV_TH_ and BA. Across different growth stages of the two tree species, biodiversity and structural diversity exhibit divergent responses.

The biodiversity affects forest plants mainly through the mechanisms of niche complementarity and the selection effect [18,19]. *C. lanceolata* exhibits divergent responses to H across different DBH classes. As DBH increases, the effect of species diversity gradually shifts from negative to positive. This is because in the early growth stage of *C. lanceolata*, the individuals are small-sized and require more sunlight for growth. Higher species diversity leads to an increase in plant density within the forest and a gradual reduction in canopy gaps [46], thus intensifying interspecific competition. In addition, other plants with well-developed root systems can absorb water from deeper soil layers during this period, and the singularity of water sources may exacerbate the intensity of competition [47,48]. Research showed that the reduction in productivity is primarily induced by of selection effect [49]. In the late growth stage of *C. lanceolata*, competition intensity weakens; accordingly, increasing species diversity can promote the biomass accumulation of *C. lanceolata*. Current practices have demonstrated that introducing broad-leaf tree species and shrubs into *C. lanceolata* pure stands can effectively increase species diversity and thus enhance forest productivity in most regions [48,50]. The results presented in Figure 6a also support this view, as the broad-leaf tree proportion (BTP) exerts a significant promoting effect on the biomass of *C. lanceolata*. This is because an appropriate proportion of broad-leaf trees can improve forest biodiversity and further enhance the efficient utilization of forest resources [3]. Furthermore, the formation of coniferous–broad-leaf mixed forests can effectively ameliorate the forest microclimate, reduce soil evaporation, and increase precipitation infiltration [51]. These improvements enable *C. lanceolata*, a shallow-rooted plant, to acquire more resources and thereby further improve its productivity, which reflects the niche complementarity effect in forest ecosystems [20].

Forest structural diversity affects tree biomass through multiple pathways, including light availability, wind dynamics, and other disturbances [17]. Current studies have demonstrated that DBH structural diversity and crown width structural diversity exert significant effects only at large spatial scales [3,30,52], Therefore, this study adopted only CV_TH_ as the indicator of structural diversity. CV_TH_ reflects the complexity of the vertical structure of forest stands. Due to resource limitations and narrow forest space, low structural diversity prevents the coexistence of species with similar functional traits, whereas species with distinct differences in functional traits can coexist [8,9,10]. This leads to intense competition among under-story and mid-story plants in the vertical structure, reducing their resource acquisition and further decreasing their biomass. In contrast, after surviving competition, over-story and mid-story plants can obtain more resources, thereby increasing their biomass more effectively [53]. The results of this study (Figure 7c,d) showed that *P. massoniana* exhibited divergent responses to CV_TH_ across different DBH classes. As DBH increased, the effect of structural diversity gradually shifted from negative to positive. Light is the most direct environmental factor affecting the survival and early growth of young trees [54]. A higher CV_TH_ may reduce the light available to under-story and mid-story plants in the forest [17,55], which explains the extremely significant negative correlation observed in Figure 7c (*p* < 0.01). The reduction in light availability further decreases the biomass of *P. massoniana* in the early growth stage, which is a consequence induced by the selection effect [3]. When *P. massoniana* grows further and reaches the over-story and mid-story layers of the forest, the complex vertical structure enables *P. massoniana* to utilize light and water resources more efficiently [56], thus facilitating higher biomass accumulation. These results are consistent with the second hypothesis of this study.

BA is defined as the ratio of the total cross-sectional area at breast height of all trees in a stand to the plot area. It serves as a direct indicator of forest growth status, as it integrates the number and size of trees and is thus directly related to tree biomass [16]. The results showed that BA exerted significant positive effects on both tree species, with larger BA corresponding to higher biomass for the two species. BA reflects the abundance of growth resources and the maturity of the forest within the plot [57]. Abundant resources can support the vigorous growth of trees, thereby enhancing the overall productivity level of the forest stand.

## 5. Conclusions

Based on natural data of 4042 coniferous trees, this study comprehensively explored the effects of biotic and abiotic factors on the biomass of *Cunninghamia lanceolata* and *Pinus massoniana*. The results indicated that screening climatic factors using the Biomod2 model could effectively improve the explanatory power of the structural equation model (SEM). *C. lanceolata* has a broader suitable planting area, and increasing biodiversity after forest establishment can enhance its productivity. In contrast, *P. massoniana* can grow on steeper slopes due to its well-developed root system, and improving structural diversity after forest establishment can promote its productivity. This study recommends that forest management should the adaptability of tree species and that mixed planting of coniferous and broad-leaved trees will contribute to the more efficient utilization of forest resources. However, this study still has several limitations. It focused on a geographical region or ecosystem with a limited spatial scale, which may restrict the general applicability of its findings. Therefore, it is necessary to expand the research to a broader spatial scale. Moreover, the low explanatory power of the SEM may be attributed to the lack of soil data, belowground biomass data, and long-term forest monitoring data. Future studies should focus on these aspects for in-depth research.

## Figures and Tables

**Table 1:** Detailed information of the study sites.

Study Site	*C. lanceolata*	*P. massoniana*
Number of sample plots	85	54
Number of trees	3029	1013
Longitude (°)	118.80–120.28	118.80–120.28
Latitude (°)	27.58–28.71	27.78–28.71
Altitude (m)	136–1141	136–667
Mean annual temperature (°C)	13.24–17.59	15.15–17.59
Mean annual precipitation (mm)	1478–1947	1478–1758

**Table 2:** The relationship between different DBH classes and biomass of *C. lanceolata* and *P. massoniana*.

Species	DBH (cm)	Average Biomass				Total Biomass CV (%)	Tree Number
		Trunk (kg)	Crown (kg)	Root (kg)	Total (kg)		
*Cunninghamia lanceolata*	5–10	9.42	4.37	4.54	18.34	38.71	1114
	11–15	25.42	10.14	11.70	47.26	24.06	907
	16–20	46.25	17.69	21.96	85.90	17.83	641
	21–25	74.14	27.44	36.11	137.69	13.80	268
	25–30	111.72	41.37	57.98	211.07	12.36	95
	>30	194.35	72.15	110.27	376.77	/	4
*Pinus massoniana*	5–10	12.21	3.81	4.18	20.21	54.06	278
	11–15	44.85	9.52	12.32	66.69	30.55	301
	16–20	86.87	16.43	23.12	126.43	18.83	196
	21–25	138.68	25.42	41.05	205.15	17.04	104
	25–30	264.89	42.56	77.21	384.67	15.73	92
	>30	481.02	74.67	155.48	711.17	/	42

**Table 3:** Component loadings and eigenvalues of principal components (PCs) chosen from PCA for environmental factors.

Species	Environmental Factors	Env PC1	Env PC2
*Cunninghamia lanceolata*	Altitude (m)	0.51	−0.18
	Slope (°)	0.10	0.32
	Aspect (°)	0.03	−0.23
	Bio 02	−0.37	−0.51
	Bio 05	−0.57	0.06
	Bio 08	−0.32	0.18
	Bio 15	−0.03	−0.71
	Bio 17	0.40	−0.12
	Cumulative proportion explained	35.82	55.95
*Pinus massoniana*	Altitude (m)	0.49	−0.29
	Slope (°)	0.24	−0.46
	Aspect (°)	0.19	0.73
	Bio 01	−0.43	0.13
	Bio 04	−0.41	−0.38
	Bio 18	0.56	0.03
	Cumulative proportion explained	48.47	70.25

## Data Availability

The datasets generated and/or analyzed during the current study are available in the Supplementary Material. The climate data (mean annual temperature and mean annual precipitation) in this study were accessed through the WorldClim database (http://www.worldclim.org/) (accessed on 6 April 2023).

## Supplementary Materials

The following supporting information can be downloaded at https://www.mdpi.com/article/10.3390/life16020277/s1. There are total of five tables and four figures in the Supplementary Material. Table S1: Biomass model table. Table S2: Environmental factors used for modeling. Table S3: Model accuracy table. Table S4: Suitable habitat area. Table S5: Model fit statistic summary of the tested SEM for carbon stocks. Figure S1: Pearson correlation analysis diagram of climatic factors. Figure S2: Natural-break classification (Jenks) map of biomass accumulation rate of *C. lanceolata* and *P. massoniana*. Figure S3: Suitable habitat distribution map of *C. lanceolata* and *P. massoniana*. Figure S4: Unoptimized structural equation model. References [58,59] are cited in the supplementary materials.
