# Supplementary material for: Explaining Productivity Differences Among Tree Species via Biotic and Abiotic Factors
Source: Life (Basel). 2026 Feb 5;16(2):277. doi: 10.3390/life16020277 (PMC12942189; doi:10.3390/life16020277)
Supplement: Supplementary file 1 [file life-16-00277-s001.zip › life-4119914-supplementary.pdf]

Supplementary material

Explaining Productivity Differences Among Tree Species via Biotic and Abiotic Factors

Liyang Tong [1,2], Kai Chen[1,2], Xiahuan Zhan [3], Kai Wang[3], Huajing Song [3], Li Ma [2] and Lijin Wang [1,*]

[1] *College of Materials and Energy Engineering, Lishui University, Lishui, Zhejiang 323000, China*

[2] *College of Agriculture and Biotechnology, Lishui University, Lishui, Zhejiang 323000, China*

[3] *Zhejiang Lishui Songyang County Environmental Monitoring Station, Lishui, Zhejiang 323400, China*

* Correspondence: lsxywlj@126.com (Lijin. Wang)

Supplementary material

S1 Supplementary Materials and Methods

S 1.1 Points Screening

The distribution data of *Cunninghamia lanceolata* and *Pinus massoniana* in Lishui were obtained from field survey data. The acquired data were then screened, with only one of the two location records retained if they fell within the same grid spatial range. Ultimately, the number of geographic locations used for model construction was 36 for *C. lanceolata* and 24 for *P. massoniana*, respectively.

S 1.2 Model Operation and Model Accuracy Assessment

The study used the Biomod2 package to construct individual models including GLM, GAM, GBM, CTA, ANN, SRE, FDA, MARS, RF, MAXENT and XGBOOST, respectively. All models were run with the default parameters of the platform model, with 10 repetitions. A total of 75% of the distribution data were set as the model training dataset, and the remaining 25% as the validation dataset [1]. After the operation was completed, selected individual models were assembled to build the ensemble model.

The evaluation indices for the accuracy assessment of the ensemble model were the area under the curve (AUC) and the true skill statistics (TSS). The AUC was calculated as an alternative measure of accuracy. Prediction accuracy is considered to be similar to random for AUC values lower than 0.5, poor for values in the range 0.5–0.7, fair in the range 0.7–0.9, and values higher than 0.9 are considered excellent. In the case of TSS, values ranging from 0.2 to 0.5 are considered poor, from 0.5 to 0.8 useful, and values larger than 0.8 are considered good to excellent [1-3]. Given the small size

of the study area, this study selected individual models meeting the criteria of TSS ≥ 0.50 and AUC ≥ 0.80 to assemble the ensemble model.

The prediction results of the ensemble model were imported into ArcGIS Pro for spatial analysis and visualization. The study adopted the natural breaks classification method (Jenks) to categorize the suitable areas of the two coniferous tree species into four classes [4], namely: unsuitable planting area ($p < 0.2$), low-suitability habitat ($0.2 \leq p < 0.4$), moderate-suitability habitat ($0.4 \leq p < 0.6$), and highly suitable planting area ($p > 0.6$). This integrated modeling and assessment method can evaluate the potential distribution of *C. lanceolata* and *P. massoniana* in Lishui City.

Table

Table S1

Biomass model table.

Tree Species Classification	Biomass Model	Reference Source
Pinaceae	$B_{Pinaceae} = B_1 + B_2 + B_3$<br>$B_1 = 0.0600\, H^{0.7934}\, D_{BH}^{1.8005}$<br>$B_2 = 0.1377\, L^{0.4052}\, D_{BH}^{1.4873}$<br>$B_3 = 0.0417\, H^{-0.0780}\, D_{BH}^{2.2618}$	[5]
Cupressaceae	$B_{Cupressaceae} = B_1 + B_2 + B_3$<br>$B_1 = 0.0647\, H^{0.8959}\, D_{BH}^{1.4880}$<br>$B_2 = 0.0971\, L^{0.0346}\, D_{BH}^{1.7814}$<br>$B_3 = 0.0617\, H^{-0.1037}\, D_{BH}^{2.1153}$	

Table Note: H represents tree height (m), D_{BH} denotes diameter at breast height (cm), and L indicates crown length (m). B_1, B_2, and B_3 correspond to the stem biomass (kg), crown biomass (kg), and root biomass (kg) of an individual tree (or bamboo), respectively.

Table S2

Environmental factors used for modeling

Variables	Description	Units	*Cunninghamia lanceolata*	*Pinus massoniana*
Bio 01	Annual Mean Temperature	℃		√
Bio 02	Mean Diurnal Range	℃	√	√
Bio 03	Isothermality	/		
Bio 04	Temperature Seasonality	%		√
Bio 05	Max Temperature of Warmest Month	℃	√	
Bio 06	Min Temperature of Coldest Month	℃		
Bio 07	Temperature Annual Range	℃		
Bio 08	Mean Temperature of Wettest Quarter	℃	√	√
Bio 09	Mean Temperature of Driest Quarter	℃		
Bio 10	Mean Temperature of Warmest Quarter	℃		
Bio 11	Mean Temperature of Coldest Quarter	℃		
Bio 12	Annual Precipitation	mm		
Bio 13	Precipitation of Wettest Month	mm		
Bio 14	Precipitation of Driest Month	mm		√
Bio 15	Precipitation Seasonality	/	√	√
Bio 16	Precipitation of Wettest Quarter	mm		
Bio 17	Precipitation of Driest Quarter	mm	√	√
Bio 18	Precipitation of Warmest Quarter	mm		√
Bio 19	Precipitation of Coldest Quarter	mm		

Table S3

Model accuracy table

Species	Model	AUC	TSS	Ensemble
Cunninghamia lanceolata	MAXENT	0.86	0.64	√
	GBM	0.88	0.57	√
	MARS	0.86	0.56	√
	SRE	0.76	0.53	
	GLM	0.83	0.49	
	FDA	0.85	0.49	
	CTA	0.78	0.50	
	XGBOOST	0.84	0.49	
	ANN	0.71	0.38	
	RF	0.82	0.37	
	**Ensemble model**	**0.99**	**0.91**	
Pinus massoniana	MAXENT	0.87	0.55	√
	GLM	0.85	0.50	√
	FDA	0.84	0.51	√
	GBM	0.83	0.25	
	RF	0.77	0.14	
	MARS	0.75	0.31	
	XGBOOST	0.70	0.19	
	CTA	0.65	0.31	
	SRE	0.65	0.29	
	**Ensemble model**	**0.96**	**0.83**	

Table S4

Suitable habitat area

Species	Total area (km^2)	Highly (km^2)	Moderately (km^2)	Poorly (km^2)
Cunninghamia lanceolata	9809.90	7925.13	800.73	1084.04
Pinus massoniana	7514.12	4113.02	1527.34	1873.76

Table S5

Model fit statistic summary of the tested SEM for carbon stocks.

Species	Model fit statistics summary	Original model	Optimized model
Cunninghamia lanceolata	CFI	1.00	1.00
	SRMR	0.01	0.01
	AIC	897.82	886.20
	χ^2 (*P*-value)	0.30	0.35
	R^2	0.27	0.28
Pinus massoniana	CFI	1.00	0.99
	SRMR	0.01	0.02
	AIC	520.03	516.63
	χ^2 (*P*-value)	0.40	0.23
	R^2	0.44	0.47

Abbreviations: CFI, comparative fit index; SRMR, standardized root mean square residual; AIC, akaike information criterion; χ^2, Chi-square test; R^2 indicates the total variation in carbon stocks.

Figure

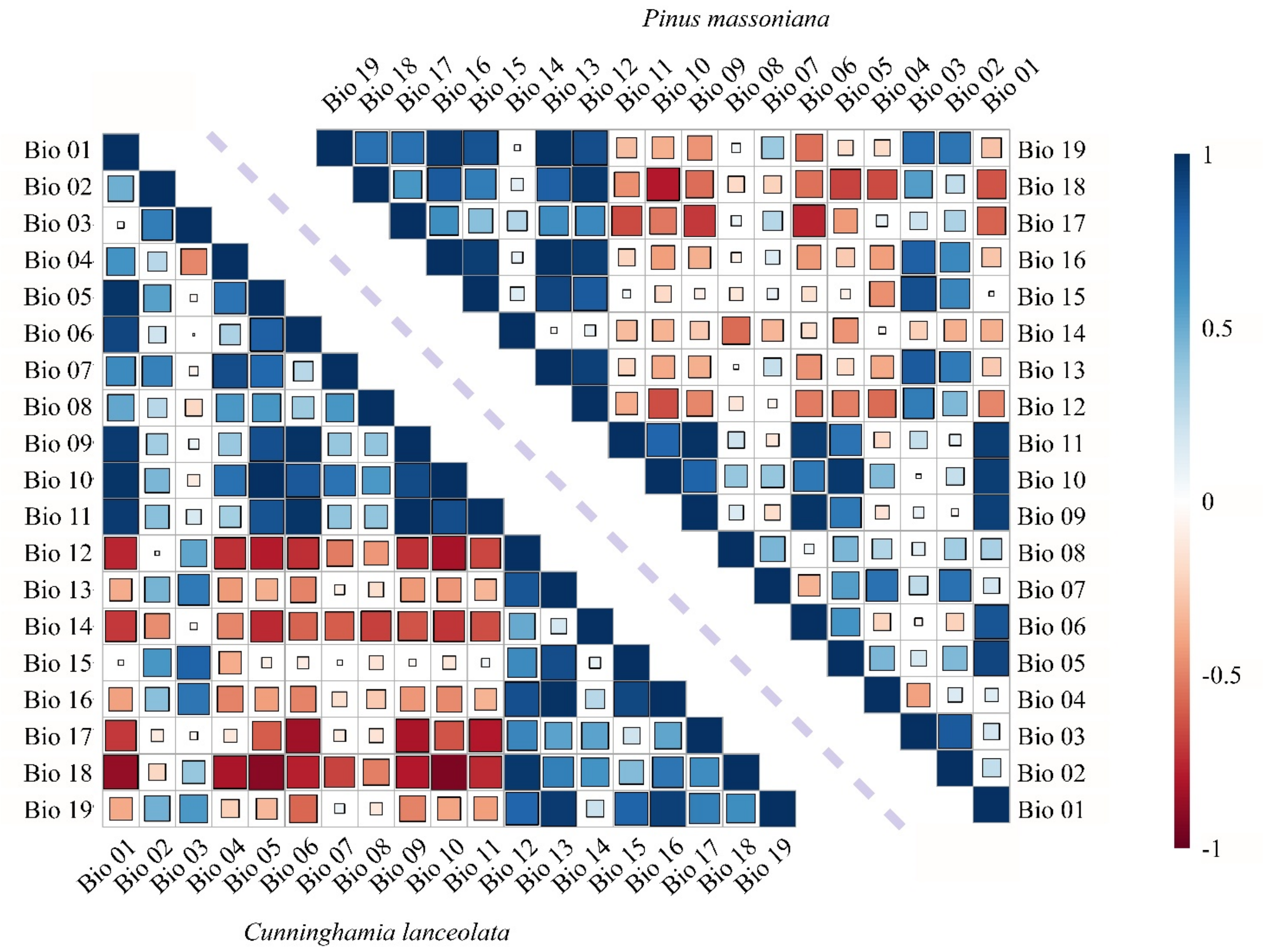

Figure S1. Pearson correlation analysis diagram of climatic factors.

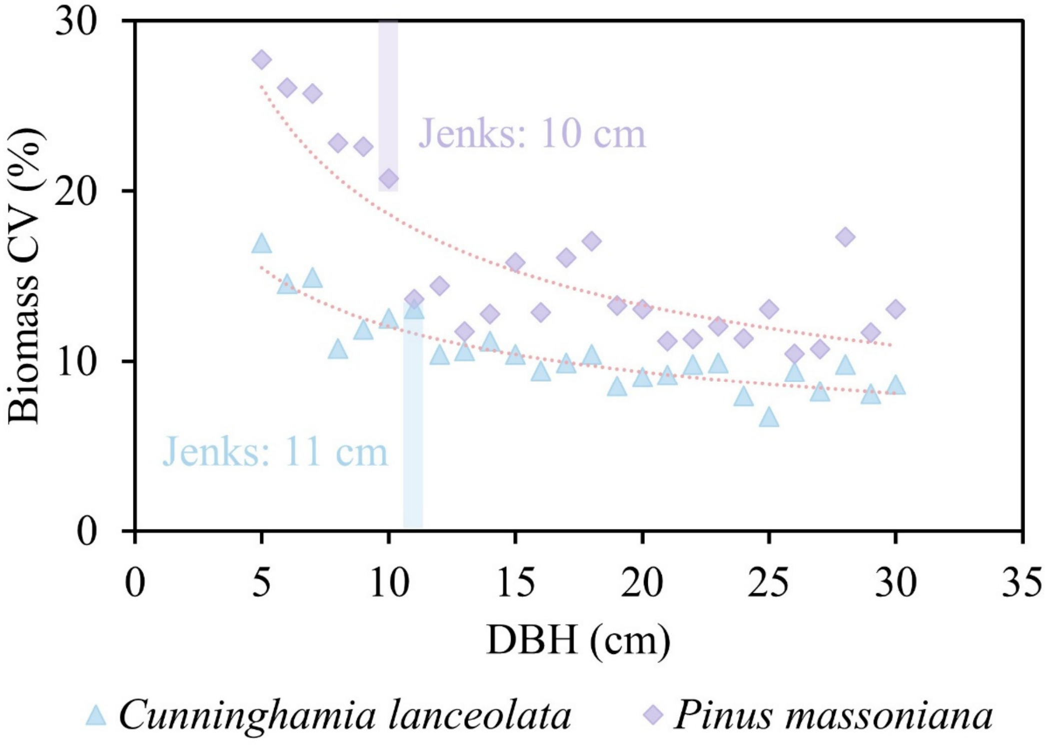

Figure S2. Natural breaks classification (Jenks) map of biomass accumulation rate of *C. lanceolata* and *P. massoniana*.

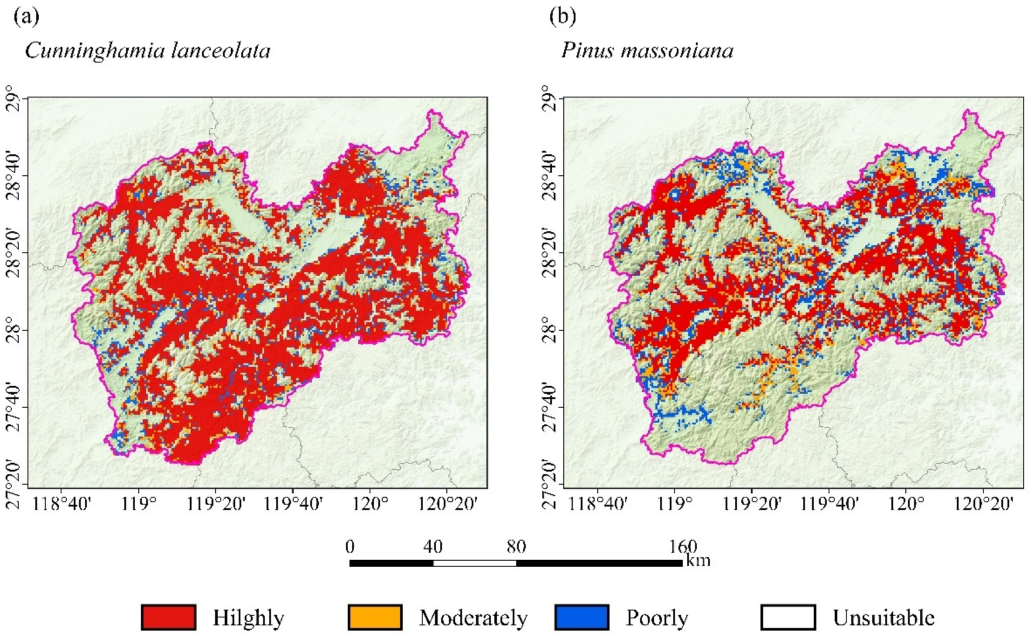

Figure S3. Suitable habitat distribution map of *C. lanceolata* and *P. massoniana*.

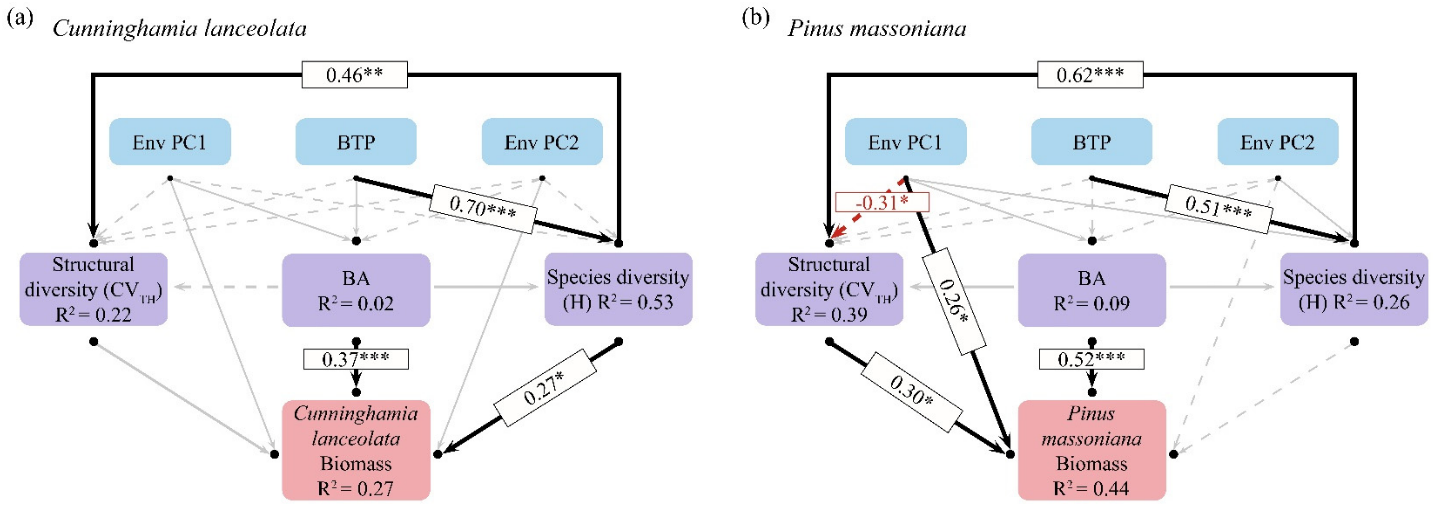

Figure S4. Unoptimized structural equation model. (a) path diagrams of factors influencing changes in *C. lanceolata* biomass, (b) path diagrams of factors influencing changes in *P. massoniana*.

References

1. Chen, K.; Ma, L.; Jiang, W.; Wang, L.; Wei, L.; Zhang, H.; Yang, R. Anthropogenic Disturbance and Climate Change Impacts on the Suitable Habitat of *Sphenomorphus incognitus* in China. *Ecol. Evol.* 2025, 15, e70848.
2. Gama, M.; Crespo, D.; Dolbeth, M.; Anastácio, P.M. Ensemble forecasting of Corbicula fluminea worldwide distribution: Projections of the impact of climate change. *Aquat. Conserv.* 2017, *27*, 675-684.
3. Coetzee, B.W.; Robertson, M.P.; Erasmus, B.F.; Van Rensburg, B.J.; Thuiller, W. Ensemble models predict Important Bird Areas in southern Africa will become less effective for conserving endemic birds under climate change. *Glob. Ecol. Biogeogr.* 2009, *18*, 701-710.
4. Chen, K.; Shao, W.; Li, Y.; Wang, L.; Lin, Z.; Guo, L.; Wei, L. Biomod2 Modeling for Predicting Suitable Distribution of Bamboo Bat (*Tylonycteris pachypus*) Under Climate Change. *Animals* 2025, 15, 1164.
5. Wang, J.; Ji, B.; Wang, Z.; Zhu, C. Influence of Subtropical Forest Landscape Pattern on Forest Carbon Density in Lishui City of Zhejiang Province. *J. Zhejiang A&F Univ.* 2024, 41, 30-40.
